# Successful Hematopoietic Stem Cell Transplantation in a Patient with Complete IFN-γ Receptor 2 Deficiency: a Case Report and Literature Review

**DOI:** 10.1007/s10875-020-00855-x

**Published:** 2020-09-10

**Authors:** Pier-Angelo Tovo, Silvia Garazzino, Francesco Saglio, Carlo Scolfaro, Jacinta Bustamante, Raffaele Badolato, Franca Fagioli

**Affiliations:** 1 Department of Public Health and Pediatric Sciences, University of Turin, Turin, Italy; 2 Department of Pediatrics, Infectious Diseases Unit, Regina Margherita Children’s Hospital, Turin, Italy; 3 Pediatric Oncohematology Division, Stem Cell Transplantation and Cell Therapy Unit, Regina Margherita Children’s Hospital, Turin, Italy; 4 Laboratory of Human Genetics of Infectious Diseases, INSERM U1163, and Center for the Study of Primary Immunodeficiencies, AP-HP, Necker Hospital for Sick Children, Paris, France; 5 Imagine Institute, University of Paris, Paris, France; 6 St. Giles Laboratory of Human Genetics of Infectious Diseases, Rockefeller Branch, The Rockfeller University, New York, NY USA; 7 Department of Pediatrics, University of Brescia, Brescia, Italy

To the Editor,

Mendelian susceptibility to mycobacterial diseases (MSMD; Online Mendelian Inheritance in Man, OMIM #209950) is an inborn error of immunity (IEI) characterized by extreme susceptibility to invasive infections sustained by poorly virulent mycobacteria, including *Mycobacterium bovis*, bacillus Calmette-Guérin (BCG) vaccines, and environmental mycobacteria [1–4]. *M. tuberculosis* may also be involved in rare cases [5]. Many genes involved in interferon (IFN)-γ production (*IL12B, IL12RB1, IL12RB2, IL23R, TYK2, ISG15, RORC*), in response to *IFN-γ (IFN-γR1, IFN-γR2, STAT1, JAK1, CYBB*), both (IRF8, SPPL2A, NEMO) or IFN-γ itself are responsible for MSDM [4–8]. The clinical features depend on the genotype and the residual activity of IFN-γ.

IFN-γR is a tetramer composed of two IFN-γR1 and two IFN-γR2 subunits. IEI in both subunits have been described and are responsible for MSMD. Autosomal recessive (AR) complete IFN-γR1 or IFN-γR2 deficiencies constitute the most severe forms of MSMD and often have fatal outcome. In these patients, the clinical manifestations begin in the first years of life and are characterized by severe, disseminated, and recurrent mycobacterial infectious diseases that require prolonged multiple antibiotic therapy [2, 3]. Exogenous IFN-γ administration is mostly ineffective when no IFN-γ response could be achieved and therefore hemopoietic stem cell transplantation (HSCT) is the only curative intervention, although a high rate of rejection has been reported in these patients [9–14]. IFNγ-R2 deficiency is rare; the gene maps to 21q22.1-22-2. From the first report [15], eleven patients from eight kindreds with AR complete IFN-γR2 deficiency have been described [16–20]. Here, we report a patient with AR complete IFN-γR2 deficiency (OMIM #147569) who had severe recurrent infections with *Mycobacterium avium* and was successfully treated with HSCT from a matched unrelated donor.

**Fig. 1:**
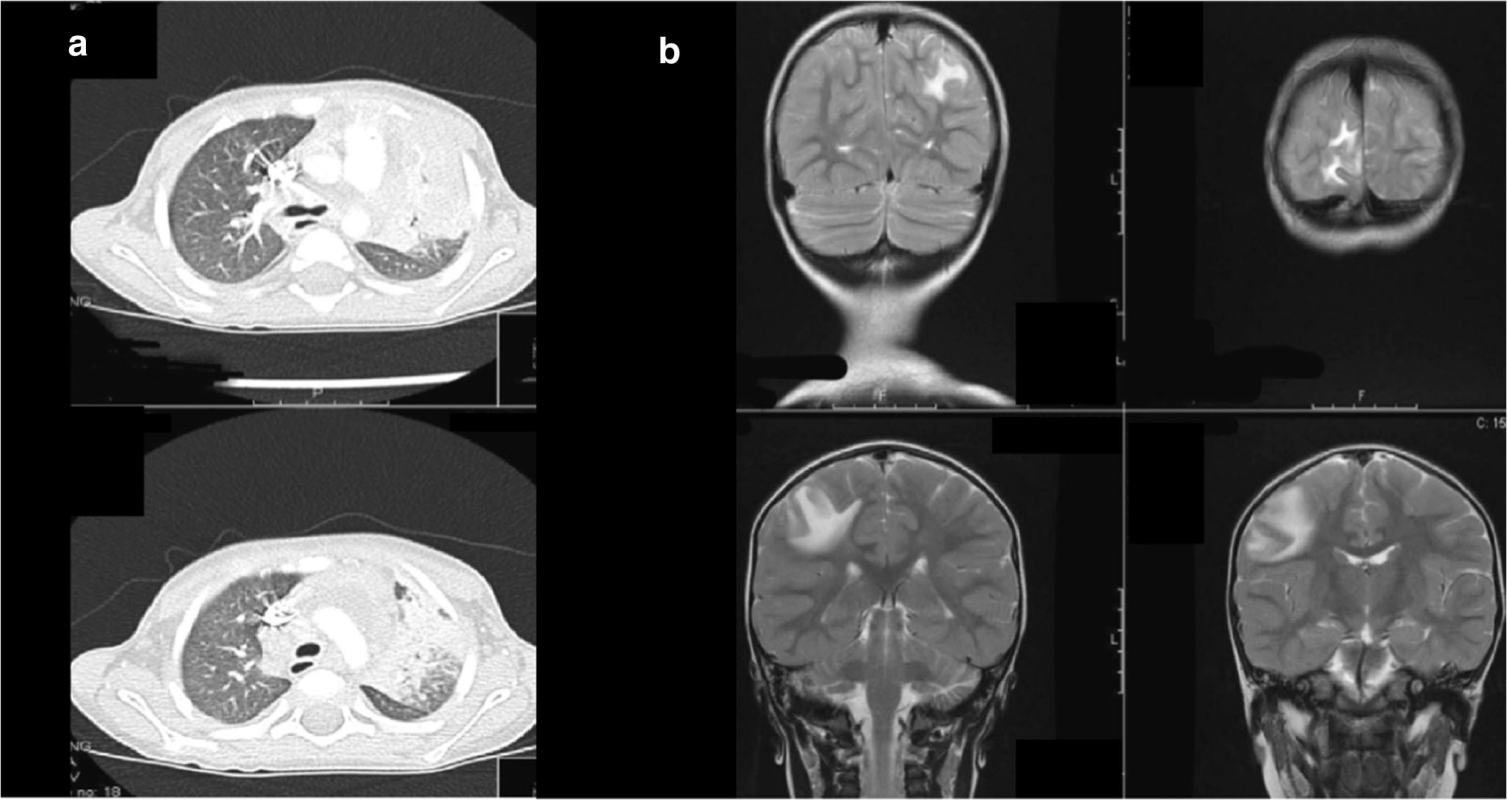
Chest CT scan of patient with mycobacterial infection and brain MRI lesions. **a** Chest CT scan: parenchymal consolidation of left upper lobe and diffuse lymphadenopathies. **b** Brain MRI: multiple hypodense cortical and subcortical lesions in the right frontoparietal and in less extent in right parietal and occipital lobes with perilesional edema

**Fig. 2:**
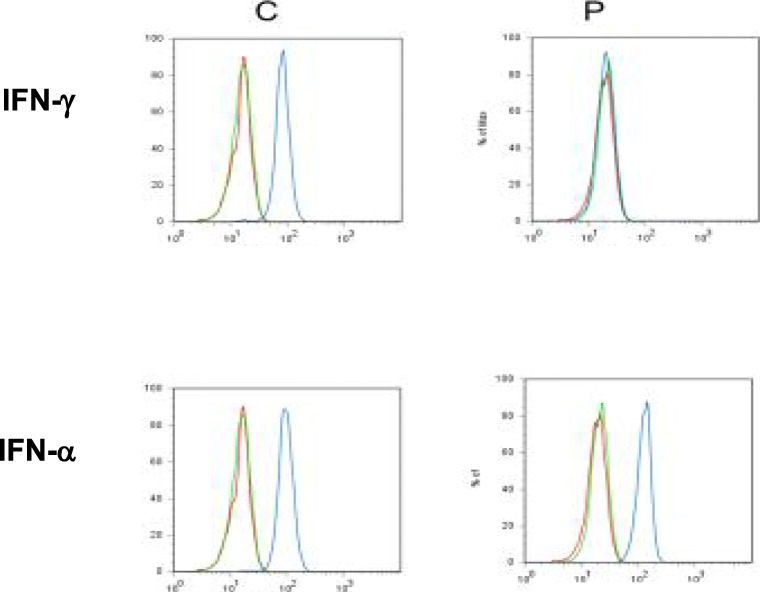
Flow cytometric analysis of STAT1 phosphorylation after stimulation of PBMCs with IFN-γ, IFN-α or medium alone. STAT1 phosphorylation in PBMCs from a control subject (C) or the patient (P) after stimulation with IFN-γ (blue lines, upper panels), IFN-α (blue lines, lower panels), or with medium alone (green lines). After cell permeabilization and fixing, cells were stained with anti-pSTAT1 or control isotype (red lines) and analyzed by flow cytometry

The child was a singleton born from unrelated Rumanian parents, after uneventful, at term pregnancy. He grew regularly and his personal history was unremarkable until 2 years of age when he had fever and cough for 1 week. Given the clinical and radiological diagnosis of left pneumonia, he was hospitalized. At physical examination, there were pallor, diffuse lymphadenopathy, and moderate hepatosplenomegaly. White blood cell (WBC) count (36,980/uL, N 68%) and inflammatory indices (C-reactive protein 156 mg/L, erythrocyte sedimentation rate 106 mm) were markedly increased. He started antibiotic therapy with intravenous ceftriaxone and then switched to meropenem and subsequent administration of teicoplanin plus azithromycin with no clinical improvement and persistently increased inflammatory markers. No microorganism was isolated in repeated cultures from blood, oropharyngeal swabs, and urines. Pulmonary computed tomography [CT] scan documented a parenchymal consolidation in the left upper lobe with diffuse lymphadenopathies suggestive of pulmonary tuberculosis (Fig. 1a), but tuberculin skin test was negative, and Quantiferon-TB Gold was indeterminate, because production of IFN-γ was observed also from cells not stimulated with MTB-complex-associated antigens (negative control tube). He did not receive BCG vaccine, and the PPD test was negative in his parents. This notwithstanding, since clinical picture and inflammatory markers did not improve, the in vitro yield of mycobacteria may require weeks, targeted investigations to exclude immunodeficiencies were in progress, and the pulmonary CT showed a typical picture of TB; 18 days later, a combined therapy for tuberculosis with isoniazid, rifampicin, ethambutol, and pyrazinamide was started without any result. After other 20 days, *Mycobacterium avium* yielded from the initial gastric lavages. Targeted therapy with ethambutol (20 mg/kg/day), clarithromycin (15 mg/kg/day), and amikacin (20 mg/kg/day) was prescribed and continued for 9 months. This led to disappearance of fever in 5 days, progressive resolution of symptoms and signs, and improvement of chest x-rays confirmed by a CT before stopping therapy. No overt abnormalities were found in routine hematological and immunological tests: HIV-negative, total number of T cells and their subpopulations, proliferative response to mitogens, and IgG levels were all within the normal ranges. Three months after suspension of therapy, the child developed a left lateralized seizure with loss of consciousness followed by winking movements at the left eye and persistent drowsiness. EEG revealed a post-critical pattern, and the neurological imaging (CT scan and resonance imaging [MRI]) showed the presence of multiple encephalic lesions mainly in the right frontoparietal and occipital lobes with significant perilesional edema (Fig. 1b). The lumbar puncture was non-contributory with only mild immunoglobulin increase and no identification of infectious agents by cultures, PCRs, or evidence of acid-fast bacilli. A cerebral biopsy was judged too risky. In the hypothesis of an encephalic spread of *M. avium,* combined treatment with clarithromycin, ethambutol, and amikacin was re-administered with addition of dexamethasone. After 2 weeks, amikacin was substituted with levofloxacin (10 mg/kg) and then with rifampicin due to persistent QT prolongation at electrocardiogram (ECG) after 4 weeks of quinolone treatment. The antimycobacterial therapy was administered for a total of 13 months without further side effects, leading to resolution of clinical manifestations, progressive reduction, and then normalization of cerebral lesions at MRI. Analysis of STAT1 phosphorylation in response to IFN-γ, IFN-α, or medium by flow cytometry showed an absence of response to IFN-γ in cells from the patient as compared with a control subject, while phosphorylation in response to IFN-α was normal (Fig. 2). Plasma level of IFN-γ was high (396 pg/ml). Taken together, all data suggested a genetic defect in IFN-γ receptor subunits. Sanger sequencing of coding exons of *IFNGR1* showed wild-type sequences, while *IFNGR2* genetic testing revealed a homozygous small deletion in exon 5 (c.663del27, predicted to lead to the in-frame deletion of residues 222–230 from IFN-γR2 protein), present in heterozygous state in his parents.

Given the poor prognosis of patients with AR complete IFN-γR deficiency, HSCT was proposed. A 22-year-old male 10 out of 10 HLA-matched unrelated donor was identified (CMV negative, blood group 0 Rh negative while patient’s blood group was B Rh positive). Bone marrow was chosen as stem cell source to reduce the risk of graft versus host disease (GvHD). When, at the age of 4 years and 4 months, the child underwent HSCT, he was in good health without any symptoms or signs of infections and normal inflammatory markers from several months. The myeloablative conditioning included iv busulfan (total dose 17.6 mg/kg), fludarabine (total dose 160 mg/kg), and thiotepa (total dose 10 mg/kg). Leviracetam prophylaxis of seizures was continued. Acyclovir, fluconazole, trimethoprim**-**sulfamethoxazole, and intravenous immunoglobulins were given as standard prophylaxis of infections. Anti-*M. avium* treatment that had been suspended for risk of toxicity was re-administered using rifampicin, ethambutol, clarithromycin, and amikacin. Given the possible enhancement of the busulfan toxicity, its concentration was monitored, but no variation of busulfan dose was required. Bone marrow infusion contained 9.7 × 10^8^ total nucleated cells/kg and 10.5 × 10^6^ CD34^+^ cells/kg. GvHD prophylaxis consisted of anti-thymocyte globulin (ATG, 7.5 mg/kg), short course of methotrexate (MTX, 10 mg/mq iv on day + 1; 8 mg/mq iv on days +3, +6 and + 11), and cyclosporine A (CycloA, 3 mg/kg iv starting day − 1 and then adjusted to achieve plasma concentrations between 100 and 250 ng/mL). The early post-transplant course was regular with no acute toxicities or severe infections. Engraftment of platelets and neutrophils were observed on day + 15 and + 18, respectively, and the patient was discharged from the bone marrow transplantation unit on day + 19. On day + 39, the control of donor-recipient chimerism on bone marrow nucleated cells showed > 97% donor’s cells. In the first 100 days after the transplantation, the patient had no signs or symptoms of GvHD; he only suffered grade 1 renal toxicities according to Bearman criteria that were treated with oral hydration and dose modulation of the main nephrotoxic drugs.

Chimerism analyses at + 6 and + 12 months confirmed complete donor’s chimerism on PBMCs. Total T cell and B cell numbers and IgG levels returned within normal ranges from +9 months (CD3 + CD4+ 423/uL; CD3 + CD8+ 128/uL; CD19+ 306/uL; IgG 942 mg/dL without replacement therapy); at that time, circulating IFN-γ level was within the normal range (< 50 pg/ml). Cyclosporine tapering was started at + 12 months with complete stop of immunosuppression 2 months later. The anti-mycobacterial drugs were progressively discontinued in parallel with the immune reconstitution, in particular amikacin was suspended at + 1, rifampicin at + 6, ethambutol at + 12 months, and clarithromycin after suspension of cyclosporine as well as levetiracetam given the normal EEG and brain MRI. At the last check, 4 years and 9 months from HSCT, the patient was in good clinical condition, and he never had signs of HSCT-related toxicities or of significant infections.

This new patient was affected by an AR complete IFN-γR2 deficiency caused by the homozygous mutation c.663del27 of exon 5. Notably, the same mutation was found in an Austrian child of consanguineous parents in whom Vogt et al. [17, 21] demonstrated that the two in-frame loss-of-function IFN-γR2 alleles encoded misfolded proteins that were abnormally N-glycosylated with a consequent complete block of the response to IFN-γ. In our patient, the underlying MSMD was responsible for the first disseminated infection with *M. avium* affecting lung, lymph nodes, liver, and spleen at 2 years of age. Combined specific antibiotic therapy for 9 months allowed the control of the infection to be reached*.* However, given the severity of the genetic defect 3 months after its suspension, there was a dramatic encephalic recrudescence. This suggests that antibiotic treatment of mycobacterial infections should be continued in these patients [2, 3]. A recent guideline provides useful recommendations for treatment of pulmonary disease caused by nontuberculous mycobacteria in adults [22], but there are no extensive studies on the prolonged use of some drugs, such as clarithromycin and quinolones, in pediatric age. To this purpose, it must be noticed that levofloxacin treatment was stopped due to QT prolongation, a rare drug-related side effect that can potentially lead to development of *torsades de pointes*, while the other anti-mycobacterial drugs, even if administered for a total of 36 months, did not elicit any adverse event. A prompt diagnosis of the immunodeficiency is however needed: in our patient, in front of the targeted suspect following the isolation of *M. avium* from gastric juice, the diagnosis was made only through in-depth genetic testing after the presumed mycobacterial infection of the brain. The child responded again to antibiotic therapy, and a compatible donor was identified.

HSCT is the only curative therapy for AR complete IFN-γR deficiency. Twelve cases with IFN-γR1 deficiency who underwent HSCT have been described: 10 from matched related donors and 2 from matched unrelated donors. High mortality and high graft rejection rate were noted among this cohort with only four successful transplantations [9–14]. Main cause of treatment failure has been ascribed to poor engraftment related to high IFN-γ serum concentrations typically found in complete IFN-γR–deficient patients [23]. High level of IFN-γ may cause cell death by Fas ligand–induced apoptosis and interfere with the cell cycles of donor HSCs [24, 25]; this negative impact could be exacerbated if donor alloreactive T cells produce IFN-γ in case of GvHD. However, at time of HSCT, our patient was in good health, with no symptoms or signs of infections and normal inflammatory markers from several months; furthermore, he did not develop any GvHD. Other five patients with AR IFN-γR2 deficiencies underwent HSCT. Two siblings received stem cells from mother and father, respectively. The older brother after full hematological engraftment developed fulminant catheter-associated *Serratia marcescens* septicemia and died, and the second son was successfully treated with paternal cells [16]. Another patient after myeloablative conditioning regimen received an unrelated umbilical cord blood unit 5/6 matched [18]. Other two siblings were successfully transplanted with haplotype-matched bone marrow from the father [20]. Therefore, all the six patients, with AR complete IFN-γR2 deficiencies, despite transitory side effects, showed a hematological reconstitution and, with the exception of the patient who died of septicemia, a stable immunological recovery. Since clinical and biologic characteristics of patients affected by AR complete IFN-γR1 or IFN-γR2 are comparable, the apparent better response in the latter might be accounted for by the small sample size or by the different therapeutic and prophylactic protocols rather than by real underlying biologic differences. The EBMT-ESID initiative collecting HSCT experiences in patients with AR IFN-yR deficiencies (https://www.ebmt.org/research/studies/hsct-experience-patients-interferon-gamma-receptor-deficiencies) may contribute to shed light on this issue and to optimize preventive and therapeutic protocols. Recently, an anti-IFN-γ monoclonal antibody (Emapalumab®) has been shown to reduce the detrimental effects of high levels of IFN-γ in patients with familial hemophagocytic lymphohistiocytosis [26], suggesting its potential use in the setting of IFN-γR deficiencies too. In our patient, possible positive aspects of the excellent response might be (a) his good general condition with no overt infection or abnormal inflammatory markers from several months before HCST; (b) the choice of a donor with 10 out of 10 HLA-matched antigens; (c) the bone marrow as stem cell source, which reduces the risk of GvHD; (d) the vigorous GvHD prophylaxis with ATG, MTX, and CycloA; (e) no sign of GvHD (and thus no associated production of IFN-γ), (f) the prophylaxis again viruses, fungi, bacteria, and peumocystosis, (g) the anti-*Mycobacterium avium* treatment until the suspension of all immunosuppressive drugs, and (h) targeted controls of possible drug toxicities through a close monitoring of organ functions and drug concentrations. The absence of any clinical manifestation at follow-up suggests that the lack of function of IFN-γR2 in non-hemopoietic tissues does not have a significant clinical impact, although this may be confirmed only by long-term post-transplant monitoring. In conclusion, this case confirms that at present, HSCT remains the only curative intervention for the block of IFN-γR signaling pathway, whereas a future alternative may be the gene therapy that in mice has been shown to be effective in restoring IFN-γR function and in protecting from mycobacterial infections [27].
